# Thromboinflammation Supports Complement Activation in Cancer Patients With COVID-19

**DOI:** 10.3389/fimmu.2021.716361

**Published:** 2021-08-18

**Authors:** Ellinor I. Peerschke, Alisa Valentino, Rachel J. So, Scott Shulman, Ravinder

**Affiliations:** Department of Laboratory Medicine, Memorial Sloan Kettering Cancer Center, New York, NY, United States

**Keywords:** COVID-19, cancer, complement, thromboinflammation, endothelial dysfunction

## Abstract

**Background:**

COVID-19 pathology is associated with exuberant inflammation, vascular damage, and activation of coagulation. In addition, complement activation has been described and is linked to disease pathology. However, few studies have been conducted in cancer patients.

**Objective:**

This study examined complement activation in response to COVID-19 in the setting of cancer associated thromboinflammation.

**Methods:**

Markers of complement activation (C3a, C5a, sC5b-9) and complement inhibitors (Factor H, C1-Inhibitor) were evaluated in plasma of cancer patients with (n=43) and without (n=43) COVID-19 and stratified based on elevated plasma D-dimer levels (>1.0 μg/ml FEU). Markers of vascular endothelial cell dysfunction and platelet activation (ICAM-1, thrombomodulin, P-selectin) as well as systemic inflammation (pentraxin-3, serum amyloid A, soluble urokinase plasminogen activator receptor) were analyzed to further evaluate the inflammatory response.

**Results:**

Increases in circulating markers of endothelial cell dysfunction, platelet activation, and systemic inflammation were noted in cancer patients with COVID-19. In contrast, complement activation increased in cancer patients with COVID-19 and elevated D-dimers. This was accompanied by decreased C1-Inhibitor levels in patients with D-dimers > 5 ug/ml FEU.

**Conclusion:**

Complement activation in cancer patients with COVID-19 is significantly increased in the setting of thromboinflammation. These findings support a link between coagulation and complement cascades in the setting of inflammation.

## Introduction

The global pandemic of coronavirus disease (COVID-19), caused by severe acute respiratory syndrome coronavirus 2 (SARS-CoV-2), is associated with significant morbidity and mortality (1). Many of the pathologic manifestations of COVID-19 are associated with an exuberant and dysregulated inflammatory response (2), resulting in the release of pro-inflammatory cytokines (3), development of coagulopathies (4), and vascular endothelial cell damage (5). In addition, complement activation (6–8) and complement deposition in vital organs on autopsy specimens (9) have been described in patients with COVID-19. Indeed, the use of pharmacologic agents targeting the complement system (10), as well as coagulation and fibrinolytic cascades (11) have been proposed.

Biomarkers of inflammation represent key prognostic indicators that may inform the selection of therapeutics in patients with COVID-19. The D-dimer, which represents a fibrin degradation product, is indicative of activation of coagulation and fibrinolysis, and elevated levels are associated with poor prognosis in patients with COVID-19 (12). A recent meta-analysis further summarizes the role of C-reactive protein, lactate dehydrogenase, and D-dimers as markers of hyperinflammation, multiorgan dysfunction, and activation of coagulation, respectively, in predicting patient outcomes in the setting of COVID-19 (13). Markers of inflammation and thrombosis, however, are associated also with cancer (14). Previous reports describing systemic markers of thromboinflammation in patients with COVID-19 have not focused on cancer patients. Since complement activation culminates in the production of anaphylatoxins C3a and C5a, as well as the C5b-9 membrane attack complex, which fuel inflammation and tissue damage (15), the current study was designed to investigate the effect of COVID-19 on circulating markers of complement activation (C3a, C5a, sC5b-9), in cancer patients with and without thromboinflammation as evidenced by plasma D-dimer levels. Selected biomarkers of vascular endothelial cell dysfunction (ICAM-1, thrombomodulin) (16, 17), and endothelial cell and platelet activation (P-selectin) (18), as well as markers of systemic inflammation linked to immunomodulation (pentraxin-3, serum amyloid A, soluble urokinase plasminogen activator (suPAR)) (19–21)were examined to inform the inflammatory response. The data suggest that complement activation is significantly enhanced in cancer patients with COVID-19 in the setting of thromboinflammation.

## Materials and Methods

### Patients and Blood Specimens

This retrospective study used clinical laboratory waste blood samples from hospitalized patients to examine the relationship between activation of coagulation and inflammation and the complement system in cancer patients with and without COVID-19. Specimens were selected from samples submitted for D-dimer analysis with enough residual plasma to support additional studies. Samples were selected based on D-dimer test results, across the reportable range from <0.5 μg/ml FEU to >20,000 μg/ml FEU, and COVID-19 status. Automated D-dimer analysis (STA Compact Max, Diagnostica Stago, S.A.S., Asnieres Sure Seine, France) was performed by the Main Hospital Clinical Hematology Laboratory using the STA-Liatest D-Di assay (Diagnostica Stago). This study was approved by the Institutional Review Board (FWA-00004998, Biospecimen Protocol 16-1547).

Specimens were obtained between April 19 to May 6, 2020. Samples were deidentified and a separate link to the patient medical record was maintained. The medical record was interrogated for selected clinical and laboratory information and SARS-CoV2 test results. Qualitative molecular SARS-CoV2 testing was performed by real-time reverse-transcriptase polymerase chain reaction (RT-PCR) on nasopharyngeal swabs in the Clinical Microbiology Laboratory using several platforms and multiple targets (N_1_, N_2_, S, ORF_1_), approved under an FDA emergency use authorization (EUA).

Due to the method of sample acquisition, specimens were obtained occasionally from the same patient on consecutive days. Duplicate samples were eliminated from the analysis so that each patient was represented once in the study. In situations where D-dimer levels increased or decreased over time, the earliest dated sample with the highest D-dimer result was included in the analysis.

Demographics of hospitalized patients testing positive (n=43) or negative (n=43) for SARS-CoV2 are summarized in **Table 1**. COVID-19 positive and negative patient cohorts were well matched for gender, age, cancer diagnosis, and D-dimer levels. Patients in both groups were diagnosied a wide range of advanced stage or metastatic solid tumors including adenocarcinoma of breast, bladder, pancreas, lung, prostate, oropharynx cancer, colon, gall bladder, appendix, rectum, and stomach, as well as a gastric neuroendocrine tumor. Hematologic malignancies included myeloma, lymphoma, acute and chronic myelogenous leukemias, as well as acute and chronic lymphocytic leukemias. Patients were under active treatment/management for primary disease, disease recurrence, or follow-up post hematopoietic stem cell transplant. Overall, 13 patients in the COVID-19 positive group were managed by Intensive Care, compared to 2 patients in the COVID-19 negative group. A slightly higher 30-day mortality was noted in the COVID-19 positive cohort. Given the small study set, evaluation of the data by disease type and/or treatment was not possible.

**Table 1:** Patient demographics.

Demographic	COVID (+) (n = 43)	COVID (-) (n = 43)
**Male (n)**	19	19
**Female (n)**	24	24
**Age (years)**		
Male	63.9	52.9
Range	17-82	5.82
Median	67	64
Female	65.9	56.08
Range	20 – 83	21-84
Median	68.5	56.5
**Ethnicity (n)**		
White	23	30
Black	9	7
Asian	5	1
Other/Unknown	6	5
**Cancer Type (n)**		
Hematologic Malignancy	21	23
Solid Tumor	17	16
Melanoma	3	0
None	2	4
**Death**		
All (Number, %)	15/43, 34.9%	10/43, 23.3%
Within 30 days (Number, %)	10/43, 23.3%	8/43, 18.6%
**D-Dimer (μg/ml FEU)**		
All	5.4 ± 6.4*	3.8 ± 5.0*
**D-Dimer (> 1.0 μg/ml FEU)**		
Positive (n)	35	30
Negative (n)	8	13

(*) p = 0.125.

### Sample Processing

Blood samples were anticoagulated with 0.0109 M (0.32%) trisodium citrate. Whole blood was centrifuged at 4000g for 5 min at room temperature to obtain platelet poor plasma, using the STAT SPIN-Express 4 centrifuge (Stat Spin Technologies, Westwood, MA). Residual plasma was stored at -30°C for 24 h after clinical testing had been completed. Samples were transferred subsequently to the research laboratory where they were thawed and aliquoted for additional studies. Sample aliquots were frozen at -80° C until use.

### Biomarker Analysis

Selected biomarkers were evaluated using enzyme linked immunosorbent assays (ELISA), according to manufacturer instructions, as indicated below.

Biomarkers of complement activation included C3a (MicroVue Complement C3a Plus EIA, Quidel, Athens, OH), C5a (MicroVue Complement C5a EIA, Quidel, Athens, OH), and sC5b-9 (MicroVue Complement SC5b-9 Plus EIA, Quidel, Athens, OH). In addition, complement inhibitors, C1-INH (MicroVue Complement C1-Inhibitor Plus EIA, Quidel, Athens, OH) and Factor H (MicroVue Complement Factor H EIA, Quidel, Athens, OH) were investigated. Since the complement system is sensitive to *in vitro* activation based on time, temperature, and anticoagulants in the absence of exogenously added inhibitors (22), baseline levels were established using blood samples from healthy male and female volunteers (n=20).

Selected biomarkers of endothelial cell dysfunction, including ICAM-1 (Quantikine ELISA, Human ICAM-1/CD54 -specific immunoassay, R&D Systems, Inc., Minneapolis, MN), and thrombomodulin (Quantikine ELISA, Human Thrombomodulin/BDCA-3 immunoassay, R&D Systems, Inc., Minneapolis, MN), as well as platelet and endothelial cell activation, P-selectin (Quantikine ELISA, Human P-Selectin/CD62P immunoassay, R&D Systems, Inc., Minneapolis, MN), were evaluated. In addition, inflammatory biomarkers such as pentraxin-3 (Human Pentraxin 3, Hycult Biotech, Uden, Netherlands), serum amyloid A (Human SAA, Hycult Biotech, Uden, Netherlands), and suPAR (suPARnostic, ViroGates, Birkerod, Denmark) were assessed.

### Statistical Evaluation

Continuous variables were expressed as median, mean, and standard deviation (S.D.). Comparisons of biomarkers between patient cohorts with and without COVID-19, in the presence or absence of positive D-dimers, were performed using Wilcoxon Rank-Sum test. Spearman rank order correlation coefficients (r_s_) were calculated to assess relationships between biomarkers. All statistical calculations were performed using GraphPad Prism (Version 9; San Diego, CA). A two tailed p value <0.05 and r_s_ of ≥ 0.30 were considered statistically significant.

## Results

### D-Dimer Analysis

Plasma D-dimer levels in hospitalized cancer patients with and without COVID-19 (**Table 1**) were comparable (p=0.125). For purposes of this study, high D-dimer levels were defined as ≥1.0 μg/ml FEU. Using this definition, 35 of 43 patients in the COVID-19 positive cohort were classified as D-dimer positive, compared to 30 of 43 patients in the COVID-19 negative cohort (**Table 1**).

### Biomarkers of Complement Activation

**Figure 1:**
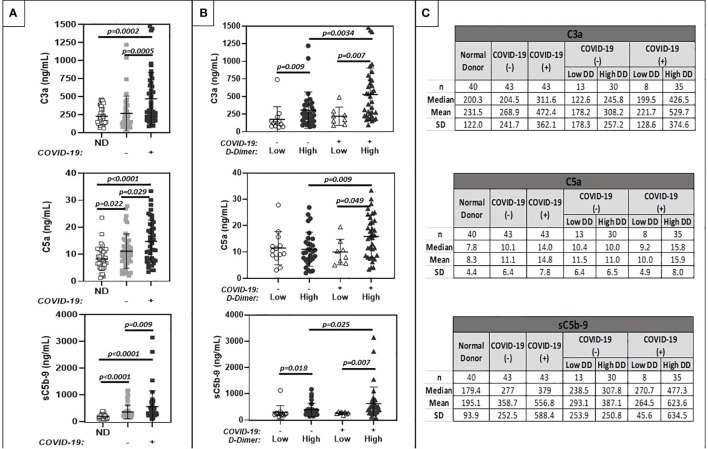
Comparison of plasma markers of systemic complement activation (C3a, C5a, sc5b-9). **(A)** shows results for normal donors (ND) (n=20), and patients with positive (+) or negative (-) COVID-19 PCR test results. **(B)** further stratifies results based on low (<1.0 ug/ml FEU) or high (>1.0 ug/ml FEU) D-dimer concentration. Numerical results are summarized in **(C)**.

Levels of complement activation markers, C3a, C5a, sC5b-9, ranged widely in plasma from hospitalized cancer patients. In patients without COVID-19, levels overlapped with those seen in healthy donors (**Figure 1A**). However, significantly elevated levels were noted in hospitalized cancer patients with COVID-19. Stratification of results by plasma D-dimer levels revealed highest levels of complement activation in patients with COVID-19 and elevated plasma D-dimer levels, and lowest levels in COVID-19 negative patients with low D-dimers (**Figure 1B**). Numerical mean and median values are summarized in **Figure 1C**. Interestingly, complement activation was similar in COVID-19 positive and negative cohorts with low D-dimers. Clinical and laboratory information accompanying specimens with markedly elevated (outlier) results were examined further, but no commonalities of diagnosis, D-dimer level, or inflammatory markers emerged.

### Complement Inhibitors

**Figure 2:**
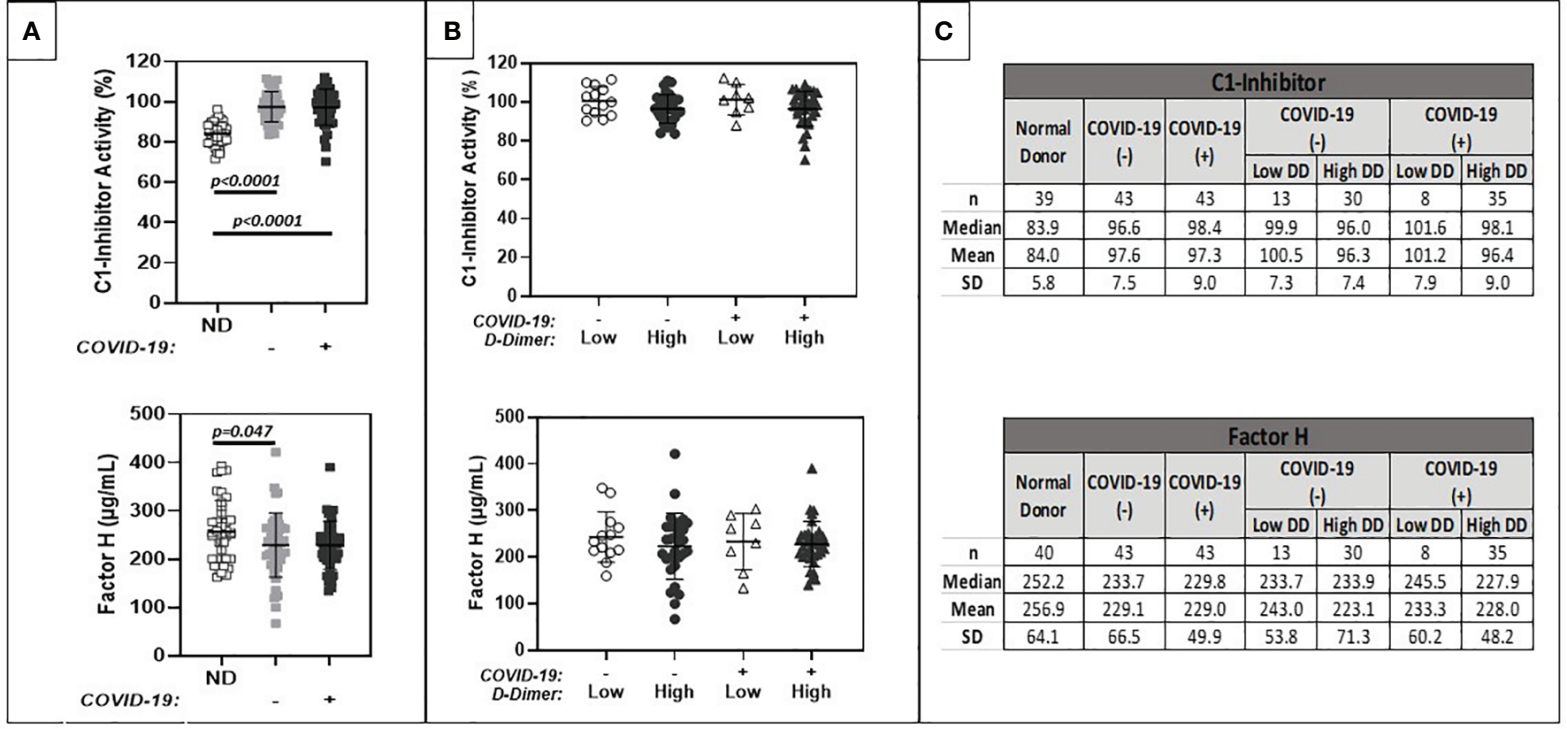
Comparison of plasma levels of circulating complement regulatory proteins (C1-INH, Factor H). **(A)** shows results for normal donors (ND) (n=20), and patients with positive (+) or negative (-) COVID-19 PCR test results. **(B)** further stratifies results based on low (<1.0 ug/ml FEU) or high (>1.0 ug/ml FEU) D-dimer concentration. Numerical results are summarized in **(C)**.

Despite evidence of systemic complement activation and activation of coagulation in cancer patient cohorts with and without COVID-19 and elevated D-dimers, C1 INH and FH levels remained within published reference ranges (Quidel MicroVue C1-Inhibitor Plus EIA. A037: 1-13; Mayo Clinic Laboratories 2020; https://www.mayocliniclabs.com/test-catalog/Clinical+and+Interpretive/64881) and were similar among all comparison groups (**Figure 2**). However, when data were stratified by D-dimer levels above and below 5 μg/ml FEU, a small (approximately 10%), but statistically significant decrease in C1 INH was noted.

### Biomarkers of Endothelial Cell Dysfunction and Platelet Activation

**Figure 3:**
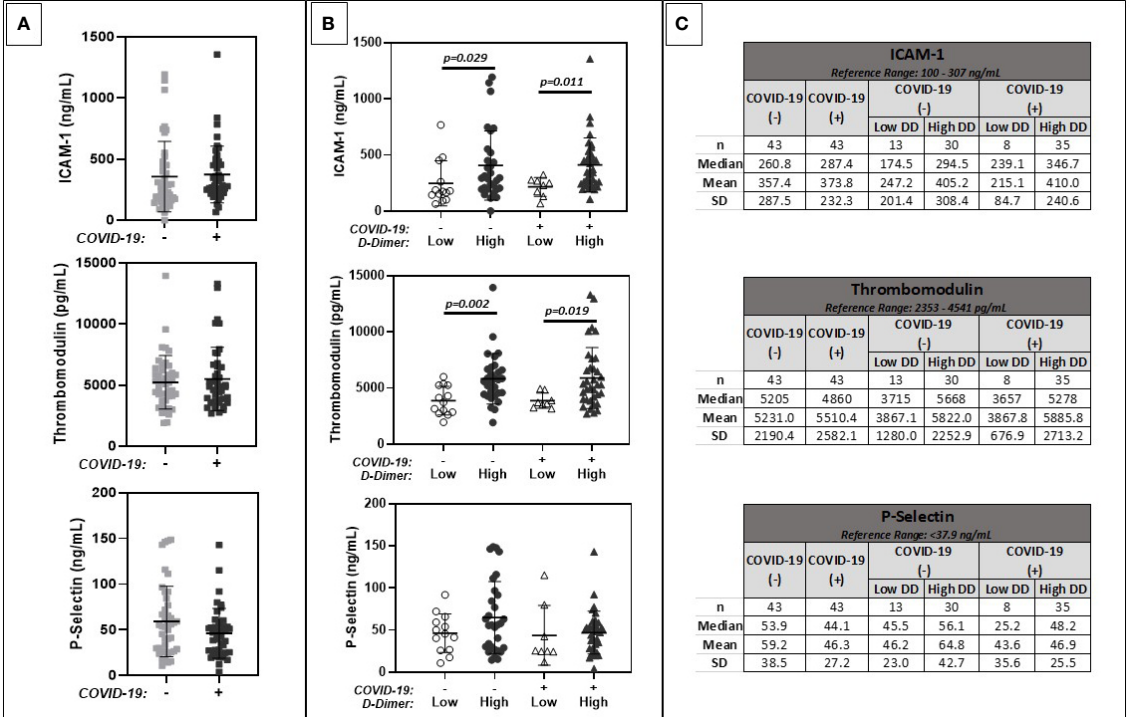
Comparison of plasma markers of endothelial cell dysfunction (ICAM 1, Thrombomodulin, P-Selectin). **(A)** shows results for patients with positive (+) or negative (-) COVID-19 PCR test results. **(B)** further stratifies results based on low (<1.0 ug/ml FEU) or high (>1.0 ug/ml FEU) D-dimer concentration. Numerical results are summarized in **(C)**.

Results are summarized in **Figure 3**. Similar levels of ICAM-1, thrombomodulin, and P-selectin levels were observed between COVID-19 positive and negative patient cohorts (Panel A). Mean levels were slightly above assay reference ranges. ICAM-1 and thrombomodulin levels trended higher in patients with positive as compared to negative D-dimers, regardless of COVID-19 positivity. Similar trends were noted for circulating P-selectin levels, but these did not reach statistical significance.

### Biomarkers of Inflammation

**Figure 4:**
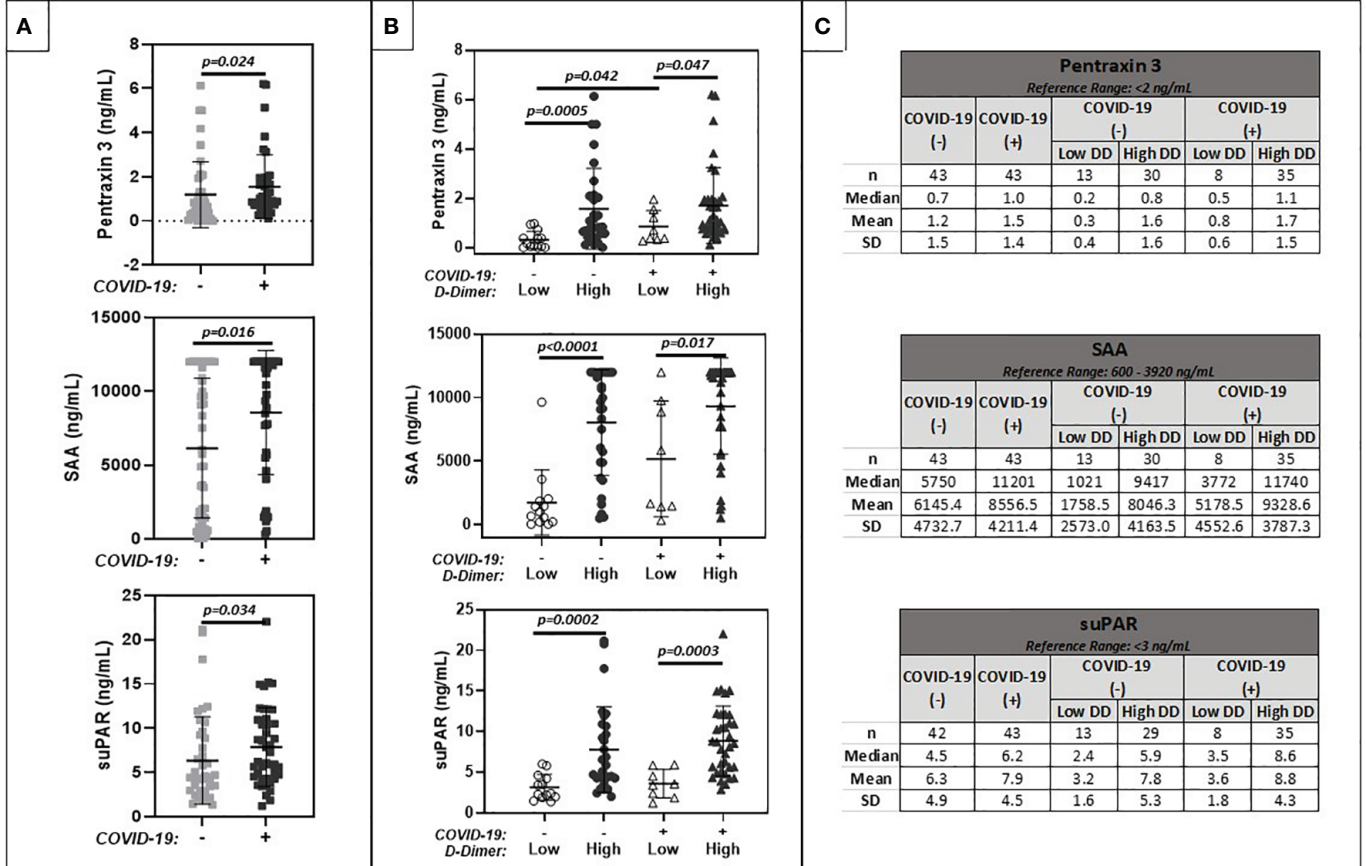
Comparison of plasma markers of inflammation (Pentraxin 3, Serum Amyloid A, suPAR). **(A)** shows results for patients with positive (+) or negative (-) COVID-19 PCR test results. **(B)** further stratifies results based on low (<1.0 ug/ml FEU) or high (>1.0 ug/ml FEU) D-dimer concentration. Numerical results are summarized in **(C)**.

Results are summarized in **Figure 4**. Levels of inflammatory markers were increased in cancer patients with COVID-19 compared to cancer patients without COVID-19. Values ranged widely among patients with and without COVID-19. Levels were highest in patients with elevated D-dimers, regardless of COVID-19 status.

### Biomarker Correlations

Correlations between biomarkers in cancer patients with or without COVID-19 were evaluated by determining Spearman rank order correlation coefficients (**Table 2**). Statistically significant correlations between complement activation, defined by elevated plasma C3a levels, and plasma D-dimers, thrombomodulin and suPAR were observed in both COVID-19 negative and positive patient cohorts. C1 INH levels correlated negatively with D-dimers. Interestingly, in the setting of COVID-19, marked complement activation was noted only in cancer patients with elevated D-dimers. D-dimer levels also correlated with markers of endothelial cell dysfunction and inflammation.

**Table 2:** Correlation between activation of coagulation (D-dimers) and complement activation (C3a) and biomarkers of endothelial cell injury and inflammation in cancer patients with or without COVID-19.

A. COVID-19 Negative.				
	D-dimer		C3a	
	rs	p	rs	p
D-dimer			0.472	0.001
C3a	0.472	0.001		
Sc5b-9	0.275	0.074	0.681	4.97E-07
C1-lnh	-0.427	0.004	-0.349	0.022
C5a	-0.013	0.935	0.129	0.408
FH	-0.100	0.523	-0.141	0.365
ICAM	0.230	0.138	0.247	0.1 10
Thombo	0.448	0.003	0.502	0.001
P-Selectin	0.224	0.149	0.331	0.030
Pentraxin	0.502	0.001	0.314	0.040
SAA	0.667	1.02E-06	0.220	0.157
suPAR	0.567	9.05E-05	0.582	5.27E-05
**B. COVID-19 Posiitve.**				
	**D-dimer**		**C3a**	
	**rs**	**p**	**rs**	**p**
D-dimer			O. 424	0.005
C3a	O. 424	0.005		
Sc5b-9	0.256	0.097	0.737	1 .80E-08
C1-lnh	-0.465	0.002	-0.097	0.535
C5a	0.220	0.156	0.557	0.0001
FH	-0 .243	0.1 16	-0.121	0.438
ICAM	0.469	0.002	0.101	0.519
Thombo	0.550	0.0001	0.486	0.001
P-Selectin	0.157	0.314	0.189	0.224
Pentraxin	0.259	0.093	0.083	0.595
SAA	0.196	0.208	-0.016	0.918
suPAR	0.703	1.49E-07	0.289	0.060

## Discussion

COVID-19 is associated with significant morbidity and mortality. Therapeutic interventions are aimed at the associated systemic inflammatory syndrome and hypercoagulability (2, 11). In addition, there is growing interest in targeting systemic complement activation (10). Systemic biomarkers may inform and optimize patient management. However, few studies have focused on cancer patients with COVID-19. Given the overlap between systemic coagulation, complement, and fibrinolytic cascades (23), this study examined selected biomarkers of complement activation, endothelial cell dysfunction and platelet activation, as well as inflammation in cancer patients in the presence or absence of COVID-19 and elevated D-dimers.

The data demonstrate that in cancer patients, D-dimer levels are associated not only with evidence of endothelial cell dysfunction and systemic inflammation but also with complement activation. In cancer patients with COVID-19, increased complement activation was noted in patients with high D-dimers (>1 μg/ml FEU). Interestingly, complement activation was similar in cancer patients with or without COVID-19 in the setting of low D-dimers. These observations suggest that complement activation during COVID-19 may be propagated by activation of coagulation and fibrinolysis, and the cross reactivity between complement and coagulation pathways (23). Consistent with this hypothesis is evidence for consumption of C1-INH, one of the major inhibitors of complement, coagulation and kinin cascades (24, 25), in cancer patients with COVID-19 and elevated D-dimers. Indeed, in the present study, C1-INH levels correlated negatively with both D-dimer levels and C3a.

Since complement activation is associated with the generation of inflammatory mediators and the potential for tissue destruction (26), complement activation may contribute to the pathology of COVID-19 and the reported increased risk of adverse outcomes in cancer patients with COVID-19 (27). Indeed, recent transcriptional profiling of nasopharyngeal swabs from patients with COVID-19 indicate that COVID-19 infection results not only in IL-6 dependent inflammatory immune responses, but also activation of complement and coagulation pathways (28), and that complement function impacts adverse COVID-19 infection outcome. Larger prospective studies are needed to further evaluate the predictive value of markers of complement activation in COVID-19.

The underlying pathophysiology of thrombosis in COVID-19 is attributed in large part to direct endothelial toxicity leading to *in situ* thrombin generation and thromboinflammation (28). Data from the present study are consistent with this concept and demonstrate that markers of endothelial cell dysfunction and inflammation were increased in the blood of cancer patients with elevated D-dimers. Since endothelial cell injury and thrombosis are hallmarks also of cancer thromboinflammation, it is not surprising that an increase in complement activation was noted in cancer patients without COVID-19 but with elevated D-dimers. Moreover, complement activation increased significantly in the setting of COVID-19 and thromboinflammation. These findings are in agreement with a recent report by Ma et al., studying non-cancer patients with respiratory failure (29), which suggests that complement activation is a general marker of critical illness, but increases the setting of COVID-19 associated respiratory failure.

These combined observations in cancer and non-cancer patients suggest a potential direct role for SARS-CoV2 mediated activation of the complement system which appear to be enhanced in the setting of thromboinflammation or critical illness. Indeed, SARS-CoV-2 activation of the lectin and alternative pathways of complement (30, 31) have been described. Moreover, the study by Ma et al. (29) demonstrates that complement activation was associated with worse outcomes in patients with COVID-19. Due to the small sample size of our study in cancer patients, we were unable to assess the impact of complement activation on disease severity and outcome.

In summary, the present study demonstrates that in cancer patients with COVID-19, D-dimer levels are associated strongly with complement activation. This study is limited by its retrospective nature and small sample size which impacts statistical power, particularly in subgroup analyses. In addition, due to retrospective sample acquisition, the available sample type and storage conditions were not optimal for analysis of complement activation products. To address this issue, complement activation assays were performed on similarly collected specimens from healthy volunteers to serve as a comparative baseline. These conditions may have influenced assay results of complement activation products, and should be considered when comparing data with other studies. Strengths of the current investigation include the focus on hospitalized cancer patients with COVID-19 and the evaluation of complement activation in the setting of cancer thromboinflammation. The present study suggests that D-dimer levels in cancer patients with COVID-19 are an indicator not only of systemic inflammation and activation of coagulation, but also enhanced complement activation. This link between inflammation, complement, and coagulation may further inform therapeutic intervention including the development of cocktails of anti-inflammatory agents, anticoagulants, and complement inhibitors. Interestingly, recent meta-analysis data suggest that therapy with the anti-inflammatory agent, tocilizumab, was associated not only with a reduction in inflammatory markers such as CRP and IL-6, but also D-dimers (32, 33). The effect on complement activation is not known at this time.

## Data Availability Statement

The original contributions presented in the study are included in the article/supplementary material. Further inquiries can be directed to the corresponding author.

## Ethics Statement

This study was approved by the Institutional Review Board/Privacy Board at Memorial Sloan Kettering (FWA-00004998, Biospecimen Protocol 16-1547). Written informed consent for participation was not required for this study in accordance with the national legislation and the institutional requirements.

## Author Contributions

EP designed the study, oversaw execution of measurements, analysis of the data, and wrote the manuscript. AV conducted measurements and analyzed the data. RS conducted measurements. SS and R provided study samples. All authors contributed to the article and approved the submitted version.

## Funding

This research was funded by in part by the NIH/NCI Cancer Center Support grant P30 CA008748.

## Conflict of Interest

The authors declare that the research was conducted in the absence of any commercial or financial relationships that could be construed as a potential conflict of interest.

## Publisher’s Note

All claims expressed in this article are solely those of the authors and do not necessarily represent those of their affiliated organizations, or those of the publisher, the editors and the reviewers. Any product that may be evaluated in this article, or claim that may be made by its manufacturer, is not guaranteed or endorsed by the publisher.
